# Influence of smoking and packaging methods on lipid stability and microbial quality of Capelin (*Mallotus villosus*) and Sardine (*Sardinella gibossa*)

**DOI:** 10.1002/fsn3.233

**Published:** 2015-04-09

**Authors:** Odoli O Cyprian, Minh Van Nguyen, Kolbrun Sveinsdottir, Asbjorn Jonsson, Tumi Tomasson, Gudjon Thorkelsson, Sigurjon Arason

**Affiliations:** 1 Department of Food Science, University of IcelandVinlandsleid 12, IS-113, Reykjavik, Iceland; 2 Kenya Marine and Fisheries Research InstituteP.O. Box 81651, Mombasa, Kenya; 3 Faculty of Food Technology, NhaTrang University02 Nguyen DinhChieu, NhaTrang, Vietnam; 4 Matís ohf./Icelandic Food and Biotech R&DVínlandsleið 12, 113, Reykjavik, Iceland; 5 United Nations University Fisheries Training programmeSkulagata 4, IS-121, Reykjavik, Iceland

**Keywords:** Capelin, fatty acids, lipid, sardine, smoked

## Abstract

Lipid and microbial quality of smoked capelin (two groups differing in lipid content) and sardine was studied, with the aim of introducing capelin in the smoked sardine markets. Lipid hydrolysis (phospholipid and free fatty acids) and oxidation index (hydroperoxides and thiobarbituric acid-reactive substances), fatty acid composition, and total viable count were measured in raw and packaged smoked fish during chilled storage (day 2, 10, 16, 22, 28). Lipid hydrolysis was more pronounced in low lipid capelin, whereas accelerated lipid oxidation occurred in high lipid capelin. Muscle lipid was less stable in sardine than capelin. Essential polyunsaturated fatty acids (eicosapentaenoic acid and docosahexaenoic acid) constituted 12% of fatty acids in capelin and 19% in sardine. Vacuum packaging as well as hot smoking retarded bacterial growth, recording counts of ≤log 5 CFU/g compared to ≥log 7CFU/g in cold smoked air packaged. Smoked low lipid capelin was considered an alternative for introduction in smoked sardine markets.

## Introduction

Fresh fish is highly perishable and various preservation techniques such as chilling, freezing, drying, salting, and smoking have been used universally to extend shelf life. In developing countries, the most affordable and widely used fish preservation methods are drying and smoking (Oduor-Odote et al. 2010; Darvishi et al. 2013). Smoking is carried out in two forms, hot and cold smoking. Hot smoking can be considered mild (30–50°C) or high temperature (50–80°C) (Marc et al. 1997), but it is commonly carried out at temperatures of 70–80°C (Erkan et al. 2011). In contrast, cold smoking is achieved without thermal treatment usually at temperatures ≤30°C (Goulas and Kontominas 2005). Smoked fish products are commonly salted. The use of salt is essential to complement the bacterial inhibitory effect of smoke by reducing water activity. For health and acceptability reasons, the practice is to have products with low salt content.

Consumption of smoked fish is increasing. In Europe, cold- and hot-smoked fish products constitute about 15% of the total fish consumption (Stołyhwo and Sikorski 2005; Huda and Dewi, 2010). The consumer preference for these products is not only for their traditionally desirable flavor but also the preservation of nutritional quality such as the highly polyunsaturated fatty acids (PUFAs) and essential amino acids (Stołyhwo et al. 2006; Bilgin et al. 2008). The long chain PUFAs, such as eicosapentaenoic acid (EPA) and docosahexaenoic acid (DHA) present in marine lipids have beneficial health effects (Stołyhwo et al. 2006; Karlsdottir et al. 2014). PUFAs are highly susceptible to oxidation, and factors such as the smoking process and storage conditions have been reported to influence their composition (Stołyhwo et al. 2006).

Smoking of small pelagic fish could be a promising processing method for species such as capelin as existing fresh markets cannot absorb the large catches for human consumption. In Iceland, capelin is one of the most important pelagic fisheries with the catch exceeding 1 million tons in some years (Hjalmarsson et al. 2010). Although small pelagic fish are mainly consumed in the dried form (Oduor-Odote et al. 2010; Darvishi et al. 2013), especially in developing countries, smoked products have also been reported (Mhongole and Mhina 2012). In Kenya, small pelagic fish are mainly used for human consumption, especially by low-income fish consumers. The demand for small pelagic fish mainly fresh water ‘dagaa’ (*Rastrineobola argentea*) has continued to rise, with declining stocks of table fish (IOC 2012). Along the Kenyan coast, the landing of marine sardines cannot meet the existing demand. At the same time, capelin is under-utilized as a food product for human consumption as most of it is used in the production of fish meal, fish oil, and pet food (Arason 2003). Supplying the market with smoked capelin could increase the direct human consumption of this small pelagic fish. Unlike in tropical sardine, the lipid content in capelin varies considerably from 3 to 4% to about 15 to 20% of their body weight with the highest lipid content in late fall (November–December) and lowest during the spawning season in March–April (Vilhjálmsson 2002). It is therefore important to determine the influence of muscle lipid content on the stability of smoked capelin with the aim of establishing appropriate harvest time for raw material.

Fish processing certainly entails a storage period for the products before consumption. Given that fish products are perishable, adequate storage conditions should be provided to slow down deteriorative changes occurring through oxidation and microbial growth (Erkan 2012). Vacuum packaging is known to inhibit oxidation and aerobic microbial growth. However, due to market demand for smoked fish that contain low quantities of smoke and salt (Beltrin et al. 1989), vacuum packaging may provide suitable conditions for *Clostridium botulinum* growth in products.

It is important for producers to have knowledge of the raw material and the effects of the processes used, so that characteristics of the finished products are consistent with market demand. The present study was aimed at determining the influence of smoking and the method of packaging on lipid hydrolysis and oxidation during refrigerated storage. A comparison between capelin caught at different times differing in lipid content and sardine was made to assess the influence of lipid content on the stability of smoked capelin, taking into consideration the possibility of its introduction as a new product into the market for smoked sardines.

## Materials and Methods

### Raw materials

Capelin samples were obtained from HB Grandi fishing company, Reykjavik, Iceland. Fish was caught on 1st (C1) and 28th (C2) February 2013 and kept in chilled seawater for 2 days prior to freezing in 25 kg blocks. One batch of sardine (S) caught on 21st March, 2013 was obtained from artisanal fisherman in Mombasa, Kenya (8 h post catch) and frozen in blocks weighing 25 kg before transportation by air freight to Iceland. Samples were kept frozen at −25°C until the time of the study.

Thawing was done overnight in open air at 18–20°C, after which twenty fish from each group were individually weighed and tagged for identification during subsequent weighing. Each group was subjected independently to strong brine infusion (brine concentration of 24% NaCl at 2°C, fish: brine ratio 1:1, w/w) for 1 h and thereafter spread on meshed trays to remove excess surface water. Each group was equally divided into two subgroups, each including 10 tagged fish for hot (H) and cold (C) smoking. The tagged samples were individually weighed prior to arranging the fish in a single layer on smoking racks.

### Smoking process

Smoking was done in a conventional smoking facility equipped with an automatic control for temperature, humidity, and density of wood smoke based on a predetermined program used for commercial production of smoked salmon. Relative humidity was kept at 50% and smoke was produced from oak in an external smouldering-type generator. Hot smoking in the kiln was divided into three stages: (1) a preliminary drying and smoking at 30°C for 2 h; (2) a drying and smoking at 40°C for 2 h; (3) cooking at 75°C for 30 min. Within each stage, the kiln was programmed to repeat cycles of drying for 5 min at air circulation of 2800 circles/min, followed by 20 min smoking, at 720 circles/min air circulation before refreshing (“killing the smoke”) for 5 min at 720 circles/min air circulation. For cold smoking, the processing time in the kiln was 4 h at 24°C in cycles of drying, smoking and refreshing as explained for the hot smoking process. During the smoking process, the temperature inside the kiln was monitored using thermometers (teste 926 thermometers AG, Germany) placed at three different location. After smoking, the products were allowed to cool at room temperature and smoking yield calculated as:

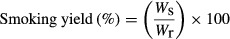


where *W*_s_ is the weight of smoked fish and *W*_r_ is the weight of brined raw material.

Smoked fish was transported to the laboratory and upon arrival 2 days postprocessing, each group was sub-sampled. The rest was further divided equally into two subgroups for air (A) and vacuum (V) packaging. About 20–30 fish were put in a high-barrier film bag (40PA/70LDPE, 250 mm × 400 mm × 0.120 mm, Plastprent, Iceland) prior to packaging using HENKOVAC packaging machine (Heavy duty 2000, Hertogenbosch, The Netherlands). Five packs per subgroup (air/vacuum) were packaged and stored at refrigerated conditions (4 ± 1°C) and samples taken for analysis on day 2, 10, 16, 22, and 28 of storage.

### Moisture content (MC), Water activity (a_w_) and salt measurements

Moisture content (MC) was determined as the weight reduction of minced fish muscle after drying at 103 ± 1°C for 4 h (ISO 1993). Results were expressed as g water/100 g muscle. Water activity (a_w_) was measured at room temperature by carefully placing about 5 g of minced sample in a clean sample cup. The sample cup was placed into a sample chamber and closed to engage the latch. Reading was complete when the instrument beeped (Novasina AW-Center, AWC503 RS-C, Axiar AG, Switzerland). The salt (NaCl) content of the samples was determined based on AOAC (2000) and expressed as g salt/100 g muscle.

### Total lipid and phospholipid content

Lipids were extracted from 25 g samples of fish muscle (80 ± 1% water) with methanol/chloroform/0.88% KCl _(aq)_ (at 1/1/0.5, v/v/v) according to the Bligh and Dyer (1959) method. The lipid content was determined gravimetrically and the results were expressed as g lipid per 100 g wet muscle.

Phospholipid content (PL) of the fish muscle was determined using a colorimetric method, based on complex formation of phospholipids and ammonium ferrothiocyanate (Stewart 1980), before reading the resultant solutions absorbance at 488 nm (UV-1800 spectrophotometer, Shimadzu, Kyoto, Japan). The reported PL was based on a standard curve prepared with phosphatidyl-choline in chloroform (5–50 *μ*g/mL) and results expressed as g PL per 100 g lipid.

### Free fatty acid (FFA)

Free fatty acid content was determined according to Bernardez et al. (2005) based on complex formation with cupric acetate-pyrimidine, followed by absorbance reading at 710 nm. The FFA concentration was calculated as *μ*mol/L quantities of oleic acid, based on a standard curve spanning a 2–22 *μ*mol range. Results were expressed as g FFA/100 g lipid.

### Lipid oxidation

Lipid hydroperoxides (PV) were determined using the ferric thiocyanate method described by Santha and Decker (1994) with modifications according to Karlsdottir et al. (2014), except that 3 ± 0.5 g of sample was used instead of 5 g, and subsequent to extraction and centrifuging at 5100 rpm for 5 min. at 4°C, 200 *μ*L of the chloroform layer was collected and mixed with 800 *μ*L of chloroform: methanol solution. The results were expressed as *μ*mol lipid hydroperoxides per kg wet muscle.

Thiobarbituric acid-reactive substances (TBARS) were measured as described by Lemon (1975) with modifications. A 3 ± 0.5 g muscle sample was homogenized with 10 mL of trichloroacetic acid (TCA) solution (7.5% TCA, 0.1% propyl gallate and 0.1% EDTA mixture prepared in distilled water), using an Ultra-Turrax homogenizer (IkaLabortechnik, T25 basic, Germany) at 8000 rpm for 10 s. The homogenate was then centrifuged at 5100 rpm for 20 min at 4°C (TJ-25 Rotor Centrifuge Beckmann, California, USA). A 100 *μ*L of supernatant was collected and mixed with 900 *μ*L of 0.02 mol/L thiobarbituric acid solution in 1.5 mL Eppendorf and heated in a water bath for 40 min at 95°C. The samples were cooled down on ice after which, 200 *μ*L was placed in duplicate into a 96-well microplate reader (NUNC A/S Thermo Fisher Scientific, Roskilde, Denmark) for absorbance reading at 530 nm (Sunrise Microplate Reader, Tecan GmbH, A-5082 Grödig, Austria). The results were expressed as *μ*mol of malomaldehyde diethylacetal/kg wet muscle and calculated based on a standard curve prepared using tetraethoxypropane.

### Fatty acids profile

The fatty acid composition was determined following derivatization of extracted TL to fatty acid methyl esters (FAME), by gas chromatography (Varian 3900 GC, Varian, Inc., Walnut Creek, CA) equipped with a fused silica capillary column (HP-88, 100 m × 0.25 mm × 0.20 *μ*m film), split injector and flame ionization detector (FID) based on AOAC (2000) programme. The result of each fatty acid was expressed as percentages of the total FAME.

### Total plate counts (TPC)

To determine microbial quality of smoked fish, aerobic plate counts (TPC) were carried out. Pooled samples were analyzed in duplicate per subgroup, observing strict hygiene to prevent cross-contamination. Twenty five grams of minced muscle were mixed with 225 mL of cooled maximum recovery diluent (MRD, Oxoid) in a stomacher bag to obtain a 10-fold dilution. Blending was done in stomacher for 1 min. Successive 10-fold dilutions were done as required. Aliquots were plated in triplicate on the plate count agar (PCA). In all cases, the pour plate technique was used. Enumeration of TPC was performed after 3 days incubation at 22°C and results expressed as log CFU/g muscle.

### Data analysis

Microsoft Excel 2010 (Microsoft Inc., Redmond, WA) was used for plotting graphs. Analysis of variance (ANOVA) was calculated using the Number Cruncher Statistical System 2000 program (NCSS Statistical Software, Kaysville, UT). The program calculates multiple comparisons, using Duncan's test to find out if sample groups differ (*P* < 0.05).

## Results and Discussions

### Chemical composition and smoking yield

Salt concentration in fish muscle was not significantly different (*P* > 0.05) between the groups and smoking methods (Table1). However, a tendency of higher NaCl concentration in hot smoked subgroups (3.48–3.87 g/100 g) compared to cold smoked subgroups (3.18–3.56 g/100 g) was observed mainly due to greater dehydration in hot smoked fish. All subgroups had a NaCl concentration above the critical level of 3% considering the minimum salt content needed to inhibit the growth of food poisoning organisms specifically *C. botulinum* (Lund and Peck 2000), while providing smoked products with an acceptable salty flavor (Cardinal et al. 2001). Water activity values ranged between 0.944 and 0.953 (Table1), which are below the critical level of 0.97 for the formation of botulinum toxin (Lund and Peck 2000). Water activity was inversely correlated with NaCl concentration whose preservative effects are ascribed to the decrease in water activity (Marc et al. 1997; Rorvik 2000). During storage, both NaCl and water activity remained apparently stable in all subgroups.

**Table 1:** Raw material water content and smoked capelin and sardine yield, salt content and water activity (A), and phospholipids data as a function of storage time based on Duncan multiple range tests (B). ± = standard deviations (*n* = 3)

A	RM water content and smoked fish yield, NaCl and aw				B	Phospholipid (g/100 g total lipid) in raw and smoked fish during storage									
	Water (g water/100 g)	Yield (%)	NaCl (g/100 g)	Water activity		Storage time (days)	2	10		16		22		28	
						RM	A	Air	Vac	Air	Vac	Air	Vac	Air	Vac
C1H	72.7 ± 0.68	76.14 ± 3.29	3.48 ± 0.46	0.949 ± 0.002		5.8 ± 0.5	4.9 ± 0.5	4.7 ± 0.4	5.0 ± 0.1	4.3 ± 0.8	4.4 ± 0.5	4.0 ± 0.9	4.5 ± 0.0^2^	4.4 ± 0.3	3.7 ± 0.2
C1C	72.7 ± 0.68	81.50 ± 2.83	3.18 ± 0.38	0.952 ± 0.001	***	5.8 ± 0.1^a^	5.2 ± 0.6^ab^	4.1 ± 0.6^b^	4.3 ± 0.7^b^	3.5 ± 0.0^c^	4.2 ± 0.7^bc^	2.3 ± 0.5^d^	2.7 ± 0.7^d^	1.7 ± 0.2^d^	1.9 ± 0.5^d^
C2H	76.9 ± 0.74	71.23 ± 2.21	3.74 ± 0.52	0.948 ± 0.000^1^	*	10.2 ± 0.2^a^	7.4 ± 0.4^b^	6.3 ± 0.3^c^	7.2 ± 0.2^b^	6.5 ± 1.1^c^	6.7 ± 0.3^c^	5.1 ± 0.1^c^	5.6 ± 0.2^c^	5.5 ± 0.3^c^	5.8 ± 0.7^c^
C2C	76.9 ± 0.74	77.10 ± 2.07	3.32 ± 0.29	0.953 ± 0.002	***	10.2 ± 0.2^a^	8.2 ± 0.5^b^	6.4 ± 0.4^c^	6.5 ± 0.6^c^	5.2 ± 0.2^d^	5.7 ± 0.8^cd^	2.0 ± 0.6^e^	2.1 ± 0.4^e^	2.0 ± 0.4^e^	2.1 ± 0.2^e^
SH	75.5 ± 1.05	70.06 ± 2.64	3.87 ± 0.44	0.944 ± 0.000	**	6.7 ± 0.0^a^	4.8 ± 0.1^b^	3.8 ± 0.0^c^	3.0 ± 0.1^c^	3.3 ± 0.7^c^	3.3 ± 0.6^c^	2.3 ± 0.4^d^	3 ± 0.0^c^	2.0 ± 0.1^d^	2.6 ± 0.3^d^
SC	75.5 ± 1.05	74.16 ± 2.70	3.56 ± 0.42	0.947 ± 0.004	**	6.7 ± 0.0^a^	5.6 ± 0.2^b^	2.9 ± 0.3^cd^	3.0 ± 0.1^c^	2.4 ± 0.4^de^	2.3 ± 0.0^e^	1.1 ± 0.3^f^	1.6 ± 0.2^f^	1.2 ± 0.2^f^	1.7 ± 0.5^f^

RM, Raw material; A, Analyses before packaging; Air, Air packaged; Vac, Vacuum packaged; C1, high-lipid capelin; C2, low-lipid capelin; S, sardine; H, hot smoked; C, cold smoked.

^1^Values equal to 0.000 are values less than 0.0005.

^2^Values equal to 0.0 are values less than 0.05.

*Significant difference at a level *P* < 0.05; **Significant difference at a level *P* < 0.01; ***Significant difference at a level *P* < 0.001. Different letters (superscript) indicate significantly different values between phospholipids samples within a row.

Preservation characteristic of smoked product is associated with the dehydration effects as well as the antimicrobial and antioxidant activity of the smoke constituents (Rorvik 2000; Goulas and Kontominas 2005). In commercial practice, processing firms invest in better technologies to control production parameters so that they maximize their gains. Processing yield is one such factor. In the present study, yield was observed to be significantly different (*P* < 0.05) between the groups and smoking methods, with hot smoked subgroups as well as low-lipid capelin (C2) and sardine (S) recording relatively low yields (Table1). This may be explained by the fact that during smoking, dehydration occurs due to the evaporation of water on the fish surface and the diffusion of water from the fish muscle to the surface. One of the factors influencing water diffusivity during smoke drying of fish is the chemical composition, particularly, the lipid content (Cardinal et al. 2001). Water diffusion was higher in the low lipid fish, resulting to high dewatering corresponding to lower yield in the groups.

### Lipid changes in smoked fish

**Figure 1:**
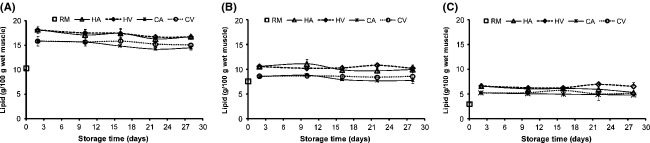
Changes in capelin (A = high lipid (C1) and B = low lipid (C2)) and sardine (C = S) muscle lipid (RM = raw material) upon smoking and chilled storage of hot-smoked (Air = HA and vacuum = HV) and cold-smoked (Air = CA and vacuum = CV) packaged samples (*n* = 3).

Lipid content in raw fish was significantly different between groups (*P* < 0.05), with values of 10.3, 7.5 and 3 g lipid/100 g muscle for C1, C2, and S groups in that order (Fig1). The capelin groups C1 and C2 caught within the same month (February) differed significantly in lipid content. The observation is in agreement with an earlier study by Arason et al. (2014) that lipid content of capelin varies considerably between and within seasons.

On smoking, an increase in lipid content was observed in all sample groups, hot smoked fish having significantly higher lipid levels than their cold-smoked counterparts (Fig.1). Increased lipid content was also influenced by the initial lipid content as the greatest increase was in fatter capelin C1. Total lipid was based on wet muscle and more water was lost during hot smoking thus increasing dry matter per unit weight. Similarly, based on the groups' initial lipid content and lipid being a constituent of dry matter, its increase was higher when smoking high lipid groups. Upon air and vacuum packaging (Fig.1), no significant change (*P* > 0.05) in lipid was observed in samples tested over storage period in respective groups. This demonstrates generally but not invariably that the total lipid remained apparently stable over the storage period in all groups. Similar results with nominal variations in total lipid were reported during drying of migaki-nishin (Shah et al. 2009) and in sardine stored in ice (Chaijan 2009).

The main lipid components in fish are triglycerides and phospholipids. Capelin groups (C1 and C2) had different initial PL content (Table1). In general, all groups had low PL proportion indicating that majority of the lipid in capelin and sardine is triglycerides. Kas'yanov et al. (2002) reported relatively high triglyceride content in capelin, with PL ratio of 11.1%, somewhat similar to the low lipid (C2) capelin (10.2 g/100 g lipid) in the present study. Since PL is a membrane lipid, it is relatively constant with minor seasonal variations (Burri et al. 2012). The low values observed in the high-lipid capelin (C1), may be accounted for by high lipid content (10.32 g/100 g muscle) in the group. On the other hand, PL obtained for raw sardine was comparatively lower than previously reported (Chaijan et al. 2006). This was probably because sardine used in the study was purchased from artisanal fishermen who did not use chilling medium on-board fishing vessels (Odoli et al. 2013). High temperatures may have led to accelerated PL hydrolysis prior to frozen storage and processing as low PL correlated well with high initial FFA content in the group.

Both smoking methods led to PL hydrolysis, particularly in the hot smoked groups (Table1). Accelerated PL hydrolysis, might be due to the greater activities of phospholipases at high temperatures (Chaijan et al. 2006; Shah et al. 2009). Phospholipid generally declined with storage time, but differently by groups (Table1). During storage, hot smoked and vacuum packaged fish had low PL hydrolytic activities compared with cold smoked and air packaged groups. Besides, low-lipid capelin (C2) and sardine (S) that had high initial PL proportion had higher PL hydrolytic activities. FFA evolution that is mainly due to lipid hydrolysis by lipolytic enzymes had greater development in C2 and sardine that had a relatively high initial PL proportion and higher PL hydrolytic activities during storage. On day 16 of storage, cold smoked fish attained low PL proportion comparable to their hot smoked counterparts on day 28, suggesting hot smoking slowed down lipolytic enzymatic activities.

**Figure 2:**
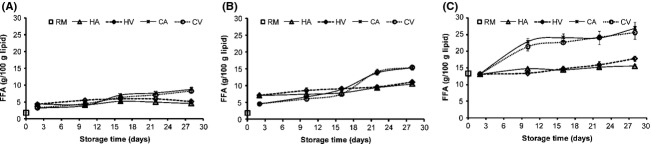
Evolution of free fatty acids in capelin (A = high lipid (C1) and B = low lipid (C2)) and sardine (C = S) muscle lipid (RM = raw material) upon smoking and chilled storage of hot smoked (Air = HA and vacuum = HV) and cold smoked (Air = CA and vacuum = CV) packaged samples (*n* = 3).

Capelin groups had lower FFA proportion in raw material than Sardines (Fig.2), indicating that hydrolysis of glycerol-fatty acid esters occurred to some extent during postmortem handling of sardine, mainly due to inappropriate handling methods (nonicing). Hydrolysis of glycerol-fatty acid esters has been reported to be an important change that occurs in fish muscle lipid postmortem with the liberation of free fatty acids (Chaijan et al. 2006). Upon smoking FFA was observed to have evolved more in hot smoked as well as fish groups that had high PL ratio. This suggests that during hot smoking, the temperature of 30–40°C for 4 h in the first two stages prior to cooking stage at 75°C for 30 min may have accelerated lipolytic enzymatic activities, which were mainly phospholipases as evolution was more in high PL groups.

During chilled storage of smoked fish, FFA content was found to evolve particularly with storage time, but progressed differently by fish groups and smoking methods. In capelin, evolution was rapid in cold smoked groups surpassing values of corresponding hot smoked groups between day 10–16 and day 16–22 for C1 and C2 accordingly. More so, the evolution was more rapid in low-lipid capelin (C2) that had higher PL. Similarly in sardines, rapid FFA evolution occurred in cold smoked groups between days 2–10 attaining 26 g/100 g lipid at the end of storage, compared to 17 g/100 g lipid for the hot smoked group. The higher FFA content in cold smoked fish can be explained by the group's high water content and lipolytic enzymatic activities that were almost stopped in the hot smoked fish. In general, FFA evolution was in agreement with the PL hydrolytic trends. Whilst, FFA had a progressive increase during storage, PL proportion decreased. It has been suggested that majority of FFA evolving in fish under refrigeration condition is derived from PL (Lopez-Amaya and Marangoni 2000). The results therefore demonstrate FFA evolution during chilled storage of smoked fish was influenced by the smoking method (more rapid in cold smoked groups) as well as the PL ratio (more rapid with high PL ratio).

### Lipid oxidation

**Figure 3:**
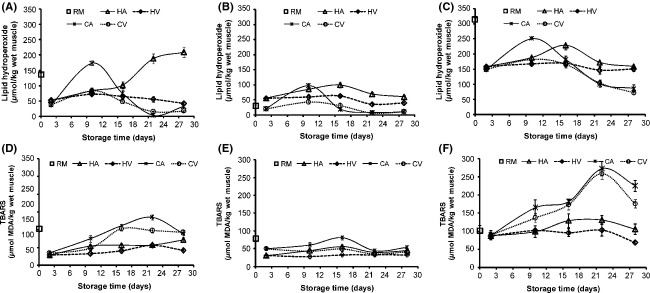
Lipid hydroperoxides formation (A, B, and C) and thiobarbituric acid-reactive substances formation (D, E, F) in capelin (high lipid (C1) = A/D; low lipid (C2) = B/E) and sardine (S = C/D) muscle lipid (RM = raw material) upon smoking and chilled storage of hot smoked (Air = HA and vacuum = HV) and cold smoked (Air = CA and vacuum = CV) packaged samples (*n* = 3).

Lipid oxidation in smoked capelin and sardine was evaluated by primary (PV) and secondary (TBARS) oxidation products (Fig.3A–F). The PV and TBARS content in raw materials was significantly different between fish groups; in capelin, high lipid group (C1) had higher values than low lipid group (C2). Unexpectedly, raw sardine obtained the highest PV value (313 *μ*mol/kg) despite its muscle constituting low lipid proportion, whereas TBARS was not statistically different from C1 (*P* > 0.05). It is likely that faster lipid oxidation occurred during postmortem handling of sardines as earlier observed with phospholipids and FFA content. Unlike phospholipids and FFA, PV and TBARS decreased in all groups except for PV in low-lipid capelin (C2) that had the least PV in raw fish. Hot smoking entails higher temperature which is generally known to accelerate lipid oxidation (Marc et al. 1997). Based on the PV and TBARS results, the influence of temperature on lipid oxidation during smoking may have been surpassed by the effects of smoke phenolic compounds with antioxidant properties (Guillen and Errecalde 2002). But temperature differences and product moisture content during hot and cold smoking, contributed to the differences in PV and TBARS obtained in the groups. Hot smoked groups were more dehydrated and thus saturated with lipid than their cold smoked counterparts.

The PV content increased in the early storage time and decreased toward the end of storage in all groups except air packaged hot smoked C1 that had progressive PV increase throughout (Fig.3A–C). Increase in PV at early storage time was due to the formation of hydroperoxides which are primary lipid oxidation products whose content depends greatly on the ratio between formation and decomposition. Lipid hydroperoxides accumulate rapidly during the initial oxidation process but with extended storage time, the rate of hydroperoxides cleavage and reactions exceed the formation (Undeland et al. 1999). In cold smoked groups a significant decrease in PV was obtained (*P* < 0.05) after day 10 and later (after day 16) in hot smoked. This indicates cold smoked fish were more vulnerable to lipid oxidation processes shown by fast accumulation and decomposition of hydroperoxides into secondary oxidation products. The vulnerability to lipid oxidation during chilled storage of cold smoked groups was in harmony with the phospholipids and FFA results. In regard to fish groups, sardine accumulated elevated PV content during early storage probably due to the high content in raw fish (Chaijan et al. 2006). Generally capelin group (C2) that had low lipid content accumulated less PV throughout the storage time, indicating the group's preference for smoking among the studied fish.

TBARS a marker for decomposition of secondary lipid oxidation products increased during the storage with a remarkable decrease at the end (Fig.3D–F). Smoked fish obtained higher TBARS content, when the groups corresponding PV content was lower (Fig.3). This as earlier explained was due to the breakdown of hydroperoxides in the later stages of lipid oxidation to secondary oxidation products. TBARS content in low-lipid capelin (C1) did not decline toward the end of storage implying delayed formation that correlated well with the group's progressive hydroperoxides accumulation observed. The decrease in TABRS after extended storage time may be explained by the malomaldehyde capability of cross-linking amino acids to form amidine linkages and/or its interactions with other components of fish which are the end products of lipid oxidation (Undeland et al. 1999). On packaging, lipid oxidation was more intense with air packaging, implying the group's susceptibility to oxidation compared to vacuum packaged. This is due to the unsaturated fatty acids reacting with molecular oxygen, usually via a free radical mechanism, to form hydroperoxides which are the primary oxidation products (Chotimarkorn et al. 2010).

### Changes in fatty acid composition

In capelin (C1 & C2) muscle lipid, the most abundant group of fatty acids was represented by monoenic acids (MUFAs) accounting for 53–55%, polyunsaturated fatty acids (PUFAs) 19–20% and saturated acids (SFAs) 19% of total fatty acids in raw fish (Table2). This is in agreement with a study by Bragadóttir et al. (2002) who reported fatty acid profile of capelin to have extraordinary high concentration of MUFAs (46–57%). In contrast sardine muscle lipid constituted SFAs as the main group, accounting for 41%, while PUFAs had a ratio of 27% and MUFAs constituted the least ratio (17%) of total FAs. Sardine muscle lipid has been reported to comprise 45.9% SFAs, 35.7% PUFAs and 16.7% MUFAs (Chaijan et al.*,* 2006), that depicts similar compositional trends to our finding but different percentage values. The difference in proportion can probably be explained by the heterogeneous nature and composition of fish fats in view that fish used in present study was obtained from a geographical different area to the allusion. Factors such as size, age, reproductive status, environmental conditions mainly water temperature can affect the fatty acid composition of fish (Saito et al. 1999).

**Table 2:** The Fatty acid composition present in the highest concentrations in the lipid extracted from the muscle of fresh and smoked capelin and sardine stored at refrigeration for 28 days. ± = standard deviations (*n* = 3)

Fish group		Storage day	Fatty Peak area of the respective FA in % of total peak area														
			C14:0	C16:0	C16:1n7	C18:0	C18:1n9	C18:1n7	C20:1n9	C18:4n3	C22:1	C20:4n6	C20:5n3	C22:6n3	∑SFA	∑MUFA	∑PUFA
C1	RM	0	6.37^a^***	11.56*	8.72***	1.03	8.47	2.75	13.51^a^**	2.16^a^***	16.17^a^**	1.83	7.23^ac^***	6.22^a^***	19.55	53.1^a^**	20.8^ac^***
	H	2	5.29^b^	13.13^a^	8.78	1.21	8.68	2.98	10.19^b^	1.93^b^	11.81^b^	1.44	9.66^b^	11.1^b^	20.3	45.6^b^	27.7^b^
		28A^1^	5.84^ab^	12.25	8.83	1.11	8.67	2.87	11.91	2.19^a^	13.03^c^	1.78	8.42^abc^	8.33^ac^	19.75	49.65^a^	24 ^cd^
		28V	5.57^ab^	12.63^ac^	8.44	1.19	8.38	2.85	11.08^b^	2.18^a^	12.97^c^	1.59	9.02^bc^	9.69^bc^	20	46.9^b^	25.95^bd^
	C	2	6.32^a^	11.43^bc^	7.94^a^	1.05	8.7	2.63	13.72^a^	2.25^a^	16.08^a^	1.87	6.96^a^	7.39^ac^	19.4	52.5^a^	21.9^ac^
		28A	6.26^ab^	11.76	9.75^b^	1.09	9.08	3.03	12.66	2.02^b^	15.18^d^	1.82	7.7^ac^	6.08^a^	19.7	53.05^a^	21^ac^
		28V	6.11^ab^	11.69	8.91	1.1	9.1	2.88	13.38^a^	1.95^b^	15.67^a^	1.89	7.46^ac^	6.52^a^	19.5	53.3^a^	21.05^ac^
C2	RM	0	6.41	11.6*	7.98	1.14	9.79*	3.03	14.03^ac^**	1.54	16.74^a^**	1.92^a^*	6.68a**	6.09^a^	19.7	55	19.3^a^***
	H	2	6.14	12.31	8.06	1.17	8.88^a^	2.97	12.1^b^	1.37	13.9^b^	1.57	8.43^a^	9.53^b^	20.2	49.2	24.1^b^
		28A	6.05	12.21	7.36	1.27	8.74^a^	2.86	12.1^b^	1.5	14.32^ab^	1.65	8.21^a^	9.88^b^	20.15	48.7	24.5^b^
		28V	5.98	11.9	7.82	1.16	8.63a	2.81	12.59^b^	1.55	14.96^ab^	1.81	8.07^a^	9.21^bd^	19.6	50.15	23.75^bc^
	C	2	6.64	10.9b	8.14	1.07	10.11^b^	3.03	14.85^a^	1.53	14.16	1.21^b^	8.25^b^	9.47^c^	19.1	51.9	23.5^c^
		28A	6.21	11.81	8.29	1.17	9.26	2.98	13.01^bc^	1.49	15.24^a^	1.81	7.88^a^	7.65^ad^	19.75	52.2	21.95^a^
		28V	6.11	11.89	8.37	1.18	9.39	3.07	12.69^b^	1.59	15.14^a^	1.79	7.89^a^	7.67^ad^	19.75	51.95	22.1^ac^
S	RM	0	3.92	24.8^ac^**	2.57^a^**	8.59**	5.85^a^**	2.26	0.56^a^**	0.47^ac^**	3.81^a^**	0.14^a^*	4.14	16.39^ab^***	40.9^ab^***	16.5^a^**	26.5**
	H	2	4.36	23.9^a^	3.44^bd^	7.45^a^	6.8^b^	2.45	2.19^b^	0.7^b^	5.1	0.31	4.81	16.27^ab^	38.9^bc^	21.7^b^	27.6^a^
		28A	4.29	23.01^b^	3.7^b^	7.64^b^	6.67^bc^	2.5	2.79^c^	0.58^c^	5.95^b^	0.43^b^	4.62	15.44^bc^	38.1^c^	23.55^c^	26.6
		28V	4.24	24.16^ab^	3.27^cd^	7.85^b^	6.29^ac^	2.32	2.03^b^	0.61^cd^	5.51^bc^	0.33	4.6	16.14^ab^	39.4^bc^	21.15^b^	27.05^a^
	C	2	4.28	24.07^ab^	3.3^cd^	7.57^b^	6.64^bc^	2.4	1.94^be^	0.66^d^	5.15^bc^	0.3	4.69	16.16^ab^	39.4^bc^	21^bd^	27.9^a^
		28A	4.25	25.08^ac^	3.02^ac^	8.9^bc^	6.47^bc^	2.4	1.56^e^	0.55^a^	4.73^ac^	0.26^a^	4.78	14.91^c^	41.7^ad^	20b^d^	26.55
		28V	4.26	26.04^c^	2.69^a^	9.57^c^	6.5^bc^	2.34	0.99^a^	0.45^a^	4.04^a^	0.23^a^	4.3	15.1^c^	43.6^d^	18.35^d^	25.2^b^

^1^Packaging (A, Air packaged; V, Vacuum packaged); RM, Raw material; C1, high-lipid capelin; C2, low lipid capelin; H, hot smoked; C, cold smoked.

*Significant difference at a level *P* < 0.05; **Significant difference at a level *P* < 0.01; ***significant difference at a level *P* < 0.001. Different letters (superscript) indicate significantly different values between samples (same group) within a column.

Among the MUFAs, erucic acid C22:1 (16% FAs) and eicosenoic acid C20:1n9 (14% FAs) were most abundant in capelin groups, whereas in sardine oleic acid C18:1n9 (6% FAs) was the major MUFAs. The SFAs were significantly higher in sardine muscle lipid and constituted palmitic acid C16:0 as predominant, with a proportion of 25% and 12% of FAs in sardine and capelin groups, respectively. There is consensus that long chain PUFAs, especially EPA (C20:5*n*-3) and DHA (C22:6*n*-3) have beneficial health outcome to consumers (Stołyhwo et al. 2006; Karlsdottir et al. 2014). Our results indicate both EPA and DHA to be present in studied fish but in varied amounts depending on the species. In capelin EPA and DHA amount were comparable with ratio of about 7% and 6%, respectively while sardine had majorly DHA constituting 16% against 4% for EPA of total FAs. Chaijan et al. (2006) found out DHA to be more abundant than EPA, with a value of 3.21 times greater. In considering health benefits of the two essential PUFAs (DHA + EPA), sardine appear to be the most valuable source of EPA + DHA. However, since these small fish are consumed as muscles not extracts and considering the lipid content of the two species in question, capelin groups (7–10 g/100 g muscle) beside sardine (3 g/100 g muscle), it is deduced that both species may be equally valuable as source of essential PUFAs.

The effects of processing and packaging technologies on fatty acid compositions of fish muscle lipid have widely been studied. Nonetheless, to our knowledge, there are no published studies on the fatty acid composition of packaged smoked capelin and sardine. Upon smoking and during storage (Table2), significant changes (*P* < 0.05) in MUFAs were observed in both capelin groups and sardine muscle lipid. SFAs remained stable in capelin groups (*P* > 0.05) but significant changes were obtained in sardine. On the contrary, MUFAs content declined in capelin groups with values corresponding to the rise in PUFAs content given that SFA appeared to be stable. In sardine MUFAs had a tendency of increasing even as PUFAs increased and SFAs content reduced. During storage, a decline on PUFAs was recorded with rapid decline in air packaged than vacuum packaged groups that related well with the formation and decomposition lipid oxidation index suggesting the losses might be due to oxidation.

### Microbial changes

**Figure 4:**
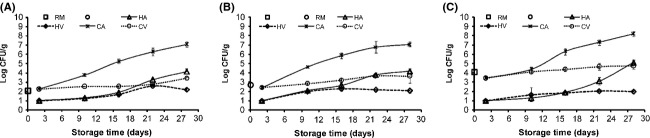
The total plate count in capelin (A = high lipid (C1) and B = low lipid (C2)) and sardine (C = S) fish (RM = raw material) upon smoking and chilled storage of hot smoked (Air = HA and vacuum = HV) and cold smoked (Air = CA and vacuum = CV) packaged samples (*n* = 3).

The aerobic plate count in raw fish was log 2 CFU/g, 2.7 CFU/g, and 4 CFU/g for C1, C2, and S in that order (Fig.4). The low bacterial count, especially in capelin groups indicate that the requirement of high initial quality of the raw material for use in fish smoking was fulfilled. After smoking, a decline in total plate count was observed in all groups but statistically significant (*P* < 0.05) with hot smoking. Total counts in capelin and sardine groups are reduced to the limit of detection (log 1 CFU/g) after hot smoking and log 2.2, 2.4, and 3.4 CFU/g in C1, C2, and S, respectively after cold smoking (Fig.4). This occurrence could be attributed to the effects of dehydration and antimicrobial activity of the smoke constituents (Rorvik 2000) besides the high temperature under hot smoking. Dehydration during smoking resulted in an increase in salt content that additionally reduced products water activity. On the other hand, high temperature (75°C for 30 min) during hot smoking may have denatured the psychotrophs that probably constituted the main microflora of defrosted fish.

During storage of packaged smoked fish, a lag phase of bacteria was observed in all groups (Fig.4), probably due to cold shock on the microbes and also the occurrence of antimicrobial smoke constituents. From day 10 of storage, high microbial growth was evident, especially in air packages. Microbial growth may be because of the diminishing intensity of antimicrobial smoke constituents, presence of oxygen, and re-establishment, or succession by cold-loving bacteria. Total counts reached log 7 CFU/g and log 4 CFU/g in cold- and hot-smoked air packaged capelin groups and log 8 CFU/g and log 5 CFU/g in cold and hot smoked sardine, respectively, on day 28. Aerobic counts of log 5 CFU/g has been used in smoked fish as limit for consumption (Hansen et al. 1995; Leroi et al. 2000). This indicates that air packaged cold smoked fish in all groups had surpassed the limit for consumption on day 28. Specifically cold smoked air packaged capelin groups reached the limit on day 16, while sardines consumed on that day surpassed by 1 log cycle. On the other hand, aerobic counts in air-packaged hot-smoked capelin groups did not reach log 5 CFU/g consumption limits, while corresponding sardine group had attained the limit on day 28.

Vacuum packaged groups showed delayed growth (*P* < 0.05) obtaining lower aerobic counts throughout the storage, indicating counts were not observed to reach log 5 CFU/g consumption limit. This could be due to the presence of low O_2_ level, retention of antimicrobial smoke constituents and to a lesser extent the low storage temperature used in the study that may have inhibited aerobic microflora development.

## Conclusions

The present study indicates accelerated lipid degradation occurred during hot smoking, but the products became more stable on chilled storage than counterpart cold smoked. Smoked capelin muscle lipid hydrolysis was more pronounced in low lipid than in high lipids, whereas accelerated lipid oxidation occurred in the high lipid group. Besides, smoked capelin muscle lipid was more stable than that of sardine. Both smoked capelin and sardine were considered a valuable source of essential PUFAs. Vacuum packaging ensured products microbial quality as well PUFAs stability and its use is recommended for smoked fish considering the right level of salt and water activity to prevent type *E C. Botulinum* poisoning. Based on oxidative stability and the fact that low-lipid capelin had higher PL proportion at the end of storage time despite accelerated hydrolytic activities, it is the preferred group for introduction as the smoked product. However, a consumer study needs to be done to ascertain products acceptability.

## References

[b1] AOAC. 2000 Fat (total, saturated, and unsaturated) in foods: method 996 06. Pp. 129–160 in Davi E, ed. Official methods of analysis of AOAC international. 17th ed. AOAC International, Gaithersburg, MD.

[b2] Arason S (2003). The drying of fish and utilization of geothermal energy: the Icelandic experience.

[b3] Arason S, Nguyen MV, Thorarinsdottir KA, Boziaris IS, Thorkelsson G (2014). Preservation of fish by curing. Seafood processing: technology, quality and safety.

[b4] Beltrin A, Peláez C, Moral A (1989). Keeping quality of vacuum-packed smoked sardine fillets: microbiological aspects. Eur. Food Res. Technol.

[b5] Bernardez M, Pastoriza L, Sampedro G, Herrera JJR, Cabo ML (2005). Modified method for the analysis of free fatty acids in fish. J. Agric. Food Chem.

[b6] Bilgin Sş, Ünlüsayin M, Izci L, Günlü A (2008). The determination of the shelf life and some nutritional components of gilthead seabream (*Sparusaurata* L., 1758) after cold and hot smoking. Turk. J. Vet. Anim. Sci.

[b7] Bligh EG, Dyer WS (1959). A rapid method of total lipid extraction and purification. Can. J. Biochem. Physiol.

[b8] Bragadóttir M, Pálmadóttir H, Kristbergsson K (2002). Seasonal changes in chemical composition and quality parameters of capelin (*Mallotus villosus*. J. Aquat. Food Prod. Tech.

[b9] Burri L, Hoem N, Banni S, Berge K (2012). Marine omega-3 phospholipids: metabolism and biological activities. J. Mol. Sci.

[b10] Cardinal M, Knockaert C, Torrissen O, Sigurgisladottir S, Mørkøre T, Thomassen M (2001). Relation of smoking parameters to the yield, colour and sensory quality of smoked Atlantic salmon (*Salmo salar*. Food Res. Int.

[b11] Chaijan M (2009). Effeects of different saturated aldehydes on the changes in sardine (*Sardinella gibbosa*) myoglobin stability. Asian J. Food Agro-Ind.

[b12] Chaijan M, Benjakul S, Visessanguan W, Faustman C (2006). Changes of lipids in sardine (*Sardinella gibbosa*) muscle during iced storage. Food Chem.

[b13] Chotimarkorn C, Silalai N, Chaitanawisuit N (2010). Changes and deterioration of lipid in farmed spotted babylon snail (Babylonia areolat a) muscle during iced storage. Food Sci. Tech. Int.

[b14] Darvishi H, Azadbakht M, Rezaeiasl A, Farhang A (2013). Drying characteristics of sardine fish dried with microwave heating. J. Saudi Soc. Agric. Sci.

[b15] Erkan N (2012). The effect of thyme and garlic oil on the preservation of vacuum-packaged hot smoked rainbow trout (*Oncorhynchus mykiss*. Food Bioprocess. Tech.

[b16] Erkan N, Ulusoy Sş, Tosun SşY (2011). Effect of combined application of plant extract and vacuum packaged treatment on the quality of hot smoked rainbow trout. J. Für. Verbrauch. Lebensm.

[b17] Goulas AE, Kontominas MG (2005). Effect of salting and smoking-method on the keeping quality of chub mackerel (*Scomber japonicus*): biochemical and sensory attributes. Food Chem.

[b18] Guillen MD, Errecalde MC (2002). Volatile components of raw and smoked black bream (Bramaraii) and rainbow trout (*Oncorhynchus mykiss*) studied by means of solid phase microextraction and gas chromatography/mass spectrometry. J. Sci. Food Agric.

[b19] Hansen LT, Gillb T, Huss HH (1995). Effects of salt and storage temperature on chemical, microbiological and sensory changes in cold-smoked salmon. Food Res. Int.

[b100] Hjalmarsson GH, Park JW, Kristbergsson K (2007). Seasonal effects on the physicochemical characteristics of fish sauce made from capelin (*Mallotus villosus*. Food Chem.

[b20] Huda N, Dewi RS (2010). Traditional smoked catfish, effects on amino acid profile. J. Fish. and Aquac. Sci.

[b21] IOC (2012).

[b22] ISO (1993). Determination of moisture and other volatile matter content (6496).

[b23] Karlsdottir GM, Sveinsdottir K, Kristinsson GH, Dominique V, Craft B, Arason S (2014). Effects of temperature during frozen storage on lipid deterioration of saithe (*Pollachius virens*) and hoki (*Macruronus novaezelandiae*. Food Chem.

[b24] Kas'yanov SP, Sayapina TA, Gor'kavaya GM, Naumenko EA, Akulin VN (2002). Correlation between the lipid composition of the Anadyr capelin *Mallotus villosus* and its physiological state. J. Evol. Biochem. Physiol.

[b25] Lemon DW (1975). Protein measurement with the Folin-Phenol reagents. J. Biol. Chem.

[b26] Leroi F, Joffraud J, Chevalier F (2000). Effect of salt and smoke on the microbiological quality of cold-smoked salmon during storage at 5 degrees C as estimated by the factorial design method. J. Food Prot.

[b27] Lopez-Amaya C, Haard FN, Simpson KB, Marangoni A (2000). Phospholipases. Seafood enzymes.

[b28] Lund B, Lund B, Baird-Parker T, Gould G, Peck M (2000). Clostridium botulinum. The microbiological safety and quality of foods.

[b29] Marc C, Kaaker R, Mboofung CM (1997). Effect of salting and smoking method on the stability of lipid and microbiological quality. J. Food Qual.

[b30] Mhongole O Mhina M 2012 Value addition in hot smoked Lake Victoria sardine (Rastrineobola argentea) for human consumption. Pp. 1–12 in IIFET, ed. Tanzania, visible possibilities: the economics of sustainable fisheries, aquaculture and seafood trade. International Institute of Fisheries Economics and Trade (IIFET), Oregon state university, USA.

[b31] Odoli CO, Oduor-Odote PM, Onyango SO (2013). Evaluation of fish handling techniques employed by artisanal fishers on quality of Lethrinus and Siganids fish genera at landing time along the Kenyan coast using sensory and microbiological methods. Afr. J. Food Agric. Nutr. Dev.

[b32] Oduor-Odote P, Shitanda D, Obiero M, Ituu G (2010). Drying characteristics and some quality attributes of *Rastrineobola argentea* (Omena) and *Stolephorus delicatulus* (kimarawali). Afr. J. Food Agric. Nutr. Dev.

[b33] Rorvik LM (2000). Listeria monocytogenes in the smoked salmon industry. Int. J. Food Micro.

[b34] Saito H, Yamashiro R, Alasalvar C, Konno T (1999). Influence of diet on fatty acids of three subtropical fish, subfamily caseioninae (*Caesio diagrama* and *C. tile*) and family siganidae (*Siganus canaliculatus*. Lipids.

[b35] Santha NC, Decker EA (1994). Rapid, sensitive, iron-based spectrophotometric methods for determination of peroxide values of food lipids. J. AOAC Int.

[b36] Shah AAKM, Tokunaga C, Kurihara H, Takahashi K (2009). Changes in lipids and their contribution to the taste of migaki-nishin (dried herring fillet) during drying. Food Chem.

[b37] Stewart JCM (1980). Colorimetric determination of phospholipids with ammonium ferrothiocyanate. Anal. Biochem.

[b38] Stołyhwo A, Sikorski ZE (2005). Polycyclic aromatic hydrocarbons in smoked fish – a critical review. Food Chem.

[b39] Stołyhwo A, Kołodziejska I, Sikorski ZE (2006). Long chain polyunsaturated fatty acids in smoked Atlantic mackerel and Baltic sprats. Food Chem.

[b40] Undeland I, Gunnar H, Lingnert H (1999). Lipid oxidation in fillets of herring (*Clupea harengus*) during ice storage. J. Agric. Food Chem.

[b41] Vilhjálmsson H (2002). Capelin (*Mallotus villosus*) in the Iceland–East Greenland–Jan Mayen ecosystem. ICES J. Mar. Sci.

